# Baseline Differences in Cochlear Implant Candidates: Bilateral Traditional vs. Expanded Indications

**DOI:** 10.3390/jcm15135068

**Published:** 2026-06-29

**Authors:** Jack Y. Lin, Andrew L. S. Thornton, Margaret L. Wilson, Barak M. Spector, Terrin N. Tamati, Aaron C. Moberly

**Affiliations:** 1 Vanderbilt University School of Medicine, Nashville, TN 37232, USA; 2 Department of Otolaryngology—Head and Neck Surgery, Vanderbilt University Medical Center, Nashville, TN 37232, USA; anthornt@utmb.edu (A.L.S.T.);; 3 Department of Speech and Hearing Science, The Ohio State University, Columbus, OH 43210, USA

**Keywords:** asymmetric hearing loss, AzBio, bilateral hearing loss, CIQOL, CNC, cochlear implant, patient-reported outcome measures, single-sided deafness, SSQ

## Abstract

**Background/Objectives:** Cochlear implant (CI) candidacy has expanded beyond traditional bilateral hearing loss (HL) to include single-sided deafness (SSD) and asymmetric hearing loss (AHL), yet baseline differences in speech recognition and patient-reported outcome measures (PROMs) between these groups—bilateral HL, SSD, and AHL—remain poorly characterized. The objective of this study was to characterize and compare preoperative speech recognition performance and PROMs between traditional bilateral HL and SSD/AHL CI candidates, and to examine associations between preoperative word recognition scores and PROMs across the full cohort. **Methods:** Sixty-eight adults (mean age 71.6 years, SD 7.4) undergoing preoperative CI evaluation were enrolled (31 bilateral HL, 12 SSD, and 25 AHL). Consonant–Nucleus–Consonant (CNC) word recognition and AzBio sentence recognition were assessed for both the ear-to-be-implanted (CI ear) and the contralateral ear. The following PROMs were evaluated: the Speech, Spatial and Qualities of Hearing Scale (SSQ-12); Cochlear Implant Quality of Life–35 (CIQOL-35); Patient Health Questionnaire-2 (PHQ-2); Tinnitus Handicap Inventory (THI); and the Instrumental Activities of Daily Living (IADL). Group comparisons used Mann–Whitney U tests and *t*-tests. CNC, SSQ-Mean, and CIQOL-Global associations were assessed using multivariable linear regression analysis. **Results:** Preoperative CI-ear speech recognition did not differ between the bilateral HL and SSD/AHL groups. SSD/AHL candidates had significantly higher contralateral-ear speech recognition performance, better SSQ-12 scores across all domains, and higher CIQOL-35 Global, Communication, Entertainment, and Environment scores compared to bilateral HL candidates. However, the CIQOL-35 Emotional, Listening Effort, and Social domains, PHQ-2, THI, and IADL did not differ significantly between the bilateral HL and SSD/AHL groups. Across our entire sample of candidates, CI-ear CNC scores were not significantly associated with preoperative SSQ-Mean or CIQOL-Global scores, while contralateral-ear CNC scores showed moderate, significant associations with both measures. **Conclusions:** Traditional bilateral and SSD/AHL CI candidates exhibit distinct preoperative PROM profiles (namely, the SSQ-12 and CIQOL-35) despite having no significant differences in CI-ear speech recognition. Contralateral-ear CNC scores—but not CI-ear scores—were significantly associated with the SSQ-Mean and CIQOL-Global, suggesting that contralateral-ear CNC scores may offer relevant insight into CI candidates’ functional hearing. These findings support population-specific counseling and highlight the complementary value of PROMs and audiometric data in CI candidacy evaluations.

## 1. Introduction

The World Health Organization (WHO) estimates that nearly 2.5 billion people worldwide will have some degree of hearing loss by 2050, with at least 700 million requiring rehabilitation services [1]. Cochlear implants (CIs) are an established and effective intervention for individuals with moderate-to-profound sensorineural hearing loss; however, despite demonstrated benefits, only an estimated 2–13% of eligible adults in the U.S. are currently CI recipients [2,3,4]. Multiple factors contribute to this device underutilization, including the cost of implantation, fear of surgery, and lack of awareness about CIs [2,5,6,7]. Additional concerns include the fear of losing residual hearing, concerns of having poor sound quality with a CI, and risk of poorer music appreciation compared to pre-implant [2,8,9]. Collectively, these concerns reflect uncertainty about the likely benefits of cochlear implantation and whether post-implant outcomes will meaningfully improve an individual’s hearing-related quality of life (HRQOL). To better understand the real-world benefits of CIs to HRQOL, patient-reported outcome measures (PROMs) have become increasingly important in the evaluation and counseling of CI candidates. Beyond traditional audiologic measures of audibility and speech recognition, PROMs can capture the lived impact of hearing loss on communication, social participation, emotional well-being, and daily functioning. Studies have also shown that baseline hearing abilities and expectations regarding post-CI HRQOL are associated with both the degree of perceived improvement and decision regret after implantation [10,11,12,13]. These findings underscore the importance of preoperative assessment of HRQOL and other patient-reported measures, preoperative counseling, and preoperative expectation management as potentially modifiable components of CI care.

Growing evidence suggests that baseline, preoperative HRQOL is an important determinant of post-implant outcomes. Studies in adult CI recipients have demonstrated that pre-CI psychosocial factors, perceived hearing difficulties, affect, and social function are associated with post-CI HRQOL and device satisfaction [10,14,15,16,17]. For example, higher and potentially unrealistic preoperative expectations have been linked to increased post-CI decision regret; in contrast, better baseline social functioning and positive affect have been associated with superior post-implant HRQOL [14,17]. Additionally, it has been shown that differences in measured pre-CI abilities (word and sentence recognition scores, as well as self-reported assessment of HRQOL) have been associated with differential improvements in post-CI outcome measures. Specifically, one study demonstrated that higher pre-CI speech recognition and HRQOL scores were associated with smaller post-CI improvements in both outcome categories when compared with those starting at a lower baseline [18]. Despite these known associations between pre-CI abilities and post-CI outcomes, there remains a limited understanding of the factors that shape baseline HRQOL and other self-reported perceptions in pre-CI candidates. Improved characterization of preoperative patient-reported experiences (e.g., baseline abilities and difficulties, tinnitus, psychosocial challenges, and perceptions of HRQOL) may therefore provide clinicians with critical context for improving pre-CI counseling and may support more realistic expectation setting, potentially improving preoperative decision making and postoperative patient satisfaction.

The need to better understand factors contributing to pre-implant, baseline PROMs has grown alongside substantial changes in CI candidacy criteria. In the 1980s, CI candidacy was restricted to individuals with bilateral, profound sensorineural hearing loss [19]. Today, not only are people with a broader range of hearing loss (moderate-to-profound) receiving CIs, but the latest 2024 guidelines for candidacy (based on the Minimum Speech Test Battery-Version 3, MSTB-3) include testing individual ears for determination of ear-specific CI candidacy [20]. Thus, CI candidacy has expanded beyond only “traditional” CI candidates with bilateral moderate-to-profound hearing loss (hereafter operationalized in this study as having aided Consonant–Nucleus–Consonant [CNC] word recognition scores of ≤60% in both ears) [21,22,23]. This expansion includes many patients who now meet CI candidacy criteria in only one ear, whether it be single-sided deafness (“SSD”: here considered as a ≤60% CNC word recognition score in one ear and normal hearing in the other ear) or asymmetric hearing losses (“AHL”: here considered as a ≤60% CNC word recognition score in one ear but >60% in the other ear) [21,22,23,24]. With this major expansion in CI candidacy criteria over time, we not only have a broader range of baseline auditory abilities in CI candidates but likely also baseline differences in self-reported functional hearing-related abilities, difficulties, and perceptions as assessed using PROMs. These known or presumed differences support the need to understand baseline abilities and experiences of different preoperative CI candidates (traditional bilateral vs. SSD/AHL) as a step towards targeted expectation setting and counseling.

To date, most published studies comparing CI patients with traditional bilateral hearing loss (HL) versus those with SSD/AHL loss have primarily focused on pre-CI and post-CI measures of hearing abilities in the audio booth (e.g., CNC scores) but not patient-reported measures [25,26,27,28,29]. For example, Jeppsen and McMurray (2025) demonstrated that post-CI SSD patients had significantly lower CNC performance in the implanted ear compared with a single implanted ear from traditional bilateral CI patients, using either the first implanted ear or the ear matched to the SSD patient’s implanted side for comparison [25]. This study controlled for age, time since activation, daily CI usage based on device datalogging, and etiology of hearing loss. Another study reported that implanted adults meeting traditional bilateral HL criteria had significantly higher monosyllabic word, phoneme, and sentence recognition scores in the implanted ear compared to implanted adults with SSD [28].

The results of these prior studies are important for characterizing outcomes using clinical measures of hearing abilities between the two populations of CI recipients, but measures of HRQOL and other PROMs likely play a complementary and pivotal role in CI patient experiences as well. Importantly, CI candidates with SSD/AHL may demonstrate relatively preserved audiologic performance (especially in bilateral testing conditions or when testing the ear contralateral to the ear to-be-implanted) while still reporting substantial listening effort, tinnitus burden, or psychosocial distress—factors not captured by speech recognition scores alone. Furthermore, published studies that focus on HRQOL have typically observed and characterized one group—either traditional bilateral or SSD/AHL—pre- and post-CI but have not directly compared the two groups [30,31]. This represents a gap in the literature, as bilateral and SSD/AHL patients are known to have different pre-CI abilities and post-CI auditory outcomes but are also likely to perceive and report differences related to HRQOL and other PROMs. Comparing the populations directly will provide clinicians with a better understanding of how pre-CI counseling might need to be tailored for each group. Critically, no prior study has directly compared preoperative baseline PROMs—including self-reported hearing abilities, HRQOL, psychosocial well-being, and tinnitus burden—between traditional bilateral HL and SSD/AHL CI candidates. This absence of direct comparison between groups at the pre-implant stage represents the core gap that the present study is designed to address.

Past studies have examined associations between post-implant CNC scores, HRQOL, and PROMs [17,32,33,34], and have shown a range of mild to high correlations among the measures. For example, McRackan et al. 2018 found the following mild correlations: HRQOL with word recognition in quiet [r = 0.276, *p* = 0.073] and sentence recognition in quiet [r = 0.204, *p* = 0.993] [34]. They also found that cochlear implant quality of life (CIQOL) showed similarly weak correlations with word recognition in quiet [r = 0.213, *p* = 0.768], word recognition in noise [r = 0.241, *p* = 0.584], and sentence recognition in noise [r = 0.255, *p* = 0.006]. On the other hand, Zhang et al. 2015 found that there was a high degree of correlation between the Spatial Hearing Questionnaire (SHQ) and Speech, Spatial and Qualities of Hearing Scale (SSQ) with word recognition at 12 months post-implantation [r = 0.752 and 0.748, respectively; *p* < 0.01 for both] [33]. However, these studies primarily studied post-implant (not pre-implant) correlations and did not account for contralateral ear (non-CI ear) performance. Given this range of reported correlation strengths between post-implant CNC scores, HRQOL, and PROMs, further evaluation of similar relationships in the pre-implant setting may be valuable. Accordingly, our study will examine the associations between pre-implant speech recognition measures and HRQOL/PROMs using linear regression models. This approach allows for estimation of how preoperative speech perception relates to patient-reported hearing experiences in CI candidates, which may inform preoperative counseling and expectation setting.

The primary objective of this study was to characterize and compare the baseline, preoperative patient-reported measures of hearing abilities, lived experiences, and HRQOL between traditional bilateral and SSD/AHL adult CI candidates. Rather than directly informing post-implant outcomes, understanding these baseline differences represents a critical first step toward developing more individualized preoperative counseling strategies. By identifying how these groups differ at baseline, clinicians may be better equipped to contextualize discussions of potential benefits and limitations of cochlear implantation. We hypothesized that traditional bilateral CI candidates would report similar baseline hearing abilities in their ear-to-be-implanted (hereafter referred to as the “CI ear”) but worse baseline hearing abilities in their non-implanted ear (“contralateral ear”) compared to SSD/AHL candidates. We also hypothesized that bilateral CI candidates would have worse functional limitations and HRQOL compared with SSD/AHL candidates, while the two groups would report no significant difference in levels of baseline psychosocial distress, tinnitus burden, and ability to perform activities of daily living.

Without prior studies directly comparing our two populations, our hypothesis about the functional limitations and HRQOL come from extrapolating pre-implant data from different studies, which show higher values for SSD/AHL users compared to bilateral HL users in SSQ-12 and CIQOL-35 subdomains [17,33,35,36,37]. Additionally, our hypothesis about the baseline psychosocial distress [38,39], tinnitus burden [40,41], and ability to perform activities of daily living [42] arises based on studies that show how each of these components may be impacted at various levels of hearing disability. In these studies, bilateral HL and SSD/AHL were never directly compared given that research comparing these groups is emerging.

A secondary objective was to evaluate the relationship between pre-implant speech recognition and PROMs. We hypothesized that CI-ear CNC scores would show a weak relationship with the CIQOL-Global and SSQ-Mean, while contralateral-ear CNC scores would show a stronger relationship with both measures, when all CI candidates were considered together. This hypothesis was grounded in the premise that the contralateral ear represents the dominant, better-functioning ear for most candidates—particularly those with SSD/AHL—and thus may directly shape how patients perceive their overall hearing abilities and HRQOL on a day-to-day basis. This premise is drawn from Sharma et al. [43], in which the authors demonstrated that hearing asymmetry in bimodal CI users systematically biases spatial hearing toward the better ear, suggesting that perceptual experience is anchored by the ear with superior hearing rather than being distributed equally across ears. This finding supports the theoretical prediction that the day-to-day self-reported hearing ability and HRQOL for CI candidates will align more closely with what the contralateral (better) ear can provide than with the severely impaired ear being considered for implantation.

## 2. Materials and Methods

### 2.1. Participants

A total of 68 adults undergoing pre-implant clinical CI evaluations were recruited from the CI audiology clinic and enrolled in the study after signing informed written consent. The participants were between the ages of 55 and 86 years (mean = 71.6, SD = 7.4). Data were collected as a part of a larger study investigating cognition, speech recognition, and PROMs in adult CI candidates and recipients at a tertiary referral CI center between January of 2025 and January of 2026. Participants were adults who were native speakers of American English and were undergoing CI evaluation for a first implant. Individuals with prior CI experience, diagnosed cognitive impairment, or retrocochlear pathologies on preoperative imaging (e.g., vestibular schwannoma) were excluded. The study was approved by the Institutional Review Board of Vanderbilt University Medical Center (protocol code #241060, approved 16 August 2024).

As described above, traditional CI candidates had bilateral moderate-to-profound hearing loss with aided CNC word recognition scores of ≤60% in both ears [21,22,23,24]. SSD candidates had a ≤60% CNC word recognition score in the CI ear and normal hearing in the contralateral ear (defined as an unaided pure-tone average of better/lower than or equal to 25 dB HL across 500, 1000, and 2000 Hz). AHL candidates demonstrated a ≤60% CNC word recognition score in the CI ear but >60% aided CNC and an unaided PTA worse/higher than 25 dB HL in the contralateral ear [21,22,23,24]. For the purposes of this study, participants with SSD and AHL and were grouped together into one SSD/AHL group. We acknowledge that SSD and AHL may differ in audiological configuration, pathogenesis, and HRQOL impact. With only 12 SSD participants, separate analyses for SSD and AHL lacked sufficient statistical power; combined analysis was therefore a pragmatic decision. Additionally, since SSD and AHL populations are expected to rely on a better-hearing contralateral ear and are being evaluated for a CI in the poorer ear, we believed there was a comparable auditory configuration distinct from traditional bilateral hearing loss.

#### Demographic Information

Participant demographic information including age, duration of hearing loss, and unaided audiometry was collected from their medical records or patient questionnaires. Socioeconomic status (SES) was calculated based on Nittrouer and Burton (2005), where occupation and education are each scored on a scale from 0 to 8 and subsequently multiplied to yield a composite SES score ranging from 0 to 64 [44]. Based on this scale, higher scores reflect a higher SES.

### 2.2. Speech Recognition Measures

Speech recognition measures, described below, were evaluated for the CI candidates using standardized audiologic tests during the clinical CI evaluation appointment. Participants’ own hearing aids (if worn and brought to the appointment) were evaluating using a probe-microphone and a Verifit II (Audioscan, ON, Canada) device with speech stimuli presented at 60 dBA and National Acoustics Laboratories Nonlinear Version 2 (NAL-N2) targets. If the participants’ own hearing aid(s) met these targets, their own hearing aid(s) were used for speech recognition testing during the CI evaluation appointment. If the participants’ own hearing aid(s) did not meet the prescribed targets, a clinic-stock aid was fit using a foam earplug (Resound Enzo or Phonak Naida) for CI evaluation testing. Participants were evaluated in both unilateral and bilateral best-aided conditions, with CI-ear and contralateral-ear scores reported below. For unilateral testing, speech recognition was assessed using the optimized hearing aid in the CI ear or contralateral ear while the other ear was occluded. The following tests were incorporated:

#### 2.2.1. Consonant–Nucleus–Consonant (CNC) Word Scores

The Consonant–Nucleus–Consonant (CNC) word test is a standardized open-set speech recognition measure used to evaluate word recognition ability in quiet [45]. The test consists of monosyllabic words structured in a consonant–vowel–consonant format presented in quiet at 60 dB SPL. Participants are asked to repeat each word they hear, and responses are scored based on the percentage of words or phonemes correctly identified. Scores are typically reported as the percent correct, with higher percentages reflecting better speech recognition performance. The CNC test is widely used in CI research and clinical CI candidacy and postoperative outcome evaluations due to its sensitivity to improvements in speech recognition following implantation. Additionally, based on the latest 2024 guidelines for candidacy (the MSTB-3), individual ear CNC testing is taking precedence in identifying CI-candidate ears during the CI evaluation [20]. Percent correct CNC words are the measure used in this study.

#### 2.2.2. AzBio Sentence Test

The AzBio Sentence test is a speech recognition test designed to evaluate sentence recognition in both quiet and noisy listening conditions [46]. The test consists of sentences spoken by multiple talkers and presented in a conversational style to better approximate real-world listening environments. Participants are instructed to repeat the sentences they hear, and scoring is based on the percentage of words correctly repeated. Testing in noise at our center is performed using ten-talker multi-talker babble at a +5 dB signal-to-noise ratio (SNR) with speech presented at 65 dB and babble noise at 60 dB. The AzBio test is commonly used in CI candidacy evaluations and postoperative outcome assessments. Results are reported as percent correct words, with higher scores indicating better speech understanding.

### 2.3. Patient-Reported Outcome Measures (PROMs)

A set of validated PROMs was collected from the participants. The following tests were implemented:

#### 2.3.1. Speech, Spatial and Qualities of Hearing Scale (SSQ-12)

The SSQ-12 is a validated short-form measure that assesses functional hearing ability with 12 items across three domains: Speech hearing, Spatial hearing, and Qualities of hearing (e.g., listening effort and sound recognition) [47,48]. Each item is rated on a 0–10 scale, with higher scores reflecting better perceived ability (i.e., less disability). Domain scores are calculated as the mean of the items within each domain, and an overall SSQ-Mean score is computed as the average of all 12 items, yielding a total score range from 0 to 10.

#### 2.3.2. Cochlear Implant Quality of Life–35 (CIQOL-35)

The CIQOL-35 is a validated instrument designed to evaluate HRQOL in individuals either using CIs or being evaluated as CI candidates across six domains: Communication, Emotional, Entertainment, Environment, Listening Effort, and Social Functioning [31,49]. The questionnaire is composed of 35 items that were selected and validated through item response theory to maximize the precision and relevance for CI users and candidates [50]. Thus, the CIQOL-35 can be applied broadly to both CI users and CI candidate populations. Responses are provided using a 5-point Likert scale, with scores then transformed to 0–100 outcome scores for each domain, with higher values indicating better functioning and quality of life. The CIQOL-Global score can also be calculated as an overall CI-related HRQOL metric using 10 items from the CIQOL-35 [49].

#### 2.3.3. Patient Health Questionnaire-2 (PHQ-2)

The Patient Health Questionnaire-2 (PHQ-2) is a brief screening instrument used to assess the presence of core depressive symptoms over the preceding two weeks [51]. The questionnaire consists of two items that evaluate the frequency of depressed mood and anhedonia. Responses are rated on a 4-point Likert scale ranging from 0 (“not at all”) to 3 (“nearly every day”). Total scores range from 0 to 6, with higher scores indicating a greater likelihood of depressive symptoms. A cutoff score of ≥3 is commonly used to identify individuals who may require further evaluation for depression using more comprehensive assessments.

#### 2.3.4. Tinnitus Handicap Inventory (THI)

The Tinnitus Handicap Inventory (THI) is a widely used self-report questionnaire designed to assess three domains related to tinnitus: the functional, emotional, and catastrophic impacts of tinnitus on daily life [52]. The instrument comprises 25 items divided into three subscales corresponding to the three domains. Each item is answered with “yes” (4 points), “sometimes” (2 points), or “no” (0 points), producing a total score ranging from 0 to 100. Higher scores indicate greater tinnitus-related handicap. Scores are commonly categorized into severity grades: slight (0–16), mild (18–36), moderate (38–56), severe (58–76), and catastrophic (78–100).

#### 2.3.5. Instrumental Activities of Daily Living (IADL)

The Lawton Instrumental Activities of Daily Living Scale (IADL) is a functional assessment tool used to evaluate an individual’s ability to perform complex daily activities necessary for independent living [53]. The scale includes eight domains: telephone use, shopping, food preparation, housekeeping, laundry, transportation, medication management, and financial management. Each domain is scored according to the individual’s level of independence, with higher scores indicating greater functional abilities. Total scores typically range from 0 (low function, dependent) to 8 (high function, independent), though scoring may vary slightly depending on the population studied.

### 2.4. Statistical Analyses

Statistical analyses were performed using RStudio (R version 4.5.1). An alpha of 0.05 was set with *p* < 0.05 used for statistical significance. In cases where data were not available for a specific comparison, the individual was excluded from that specific test.

Mann–Whitney U tests and independent samples *t*-tests were used to compare the demographical differences between the traditional bilateral HL and SSD/AHL groups for age, duration of hearing loss, and SES. The Mann–Whitney U test was performed when the data did not demonstrate a normal distribution, while the independent samples *t*-test was performed with data that demonstrated a normal distribution. The Shapiro–Wilk test was performed to determine normality in cases of uncertainty, with a normal distribution defined where *p* > 0.05 and non-normal distribution defined where *p* < 0.05. The test statistics and effect sizes were also included, depending on which test was being performed. A chi-square test was performed to compare the sex distributions of the two groups.

Similarly, Mann–Whitney U tests and independent samples *t*-tests were used to compare SSD/AHL and traditional bilateral pre-CI candidates scores in PROMs, CNC word scores, and AzBio scores. The same methodology used for the demographic comparisons was employed for assessing the normality of the data distribution and choosing the statistical test for these comparisons. Holm–Bonferroni correction was applied for multiple comparisons; original *p*-values are reported and interpreted for significance in the text below.

Finally, two multivariable linear regression analyses were performed, this time grouping together the entire cohort of 68 participants. The first analysis examined the SSQ-Mean as the outcome, with aided CNC scores in the CI ear and the contralateral ear tested as two separate predictors, while accounting for demographic variables of age, duration of hearing loss, and SES as additional predictors. The second analysis was the same but with the CIQOL-Global as the outcome. These two regression analyses were performed to determine the degree to which CNC scores in the CI ear and contralateral ear would relate to these two broader PROMs of perceived hearing ability (SSQ-Mean) and HRQOL (CIQOL-Global). Variance Inflation Factors (VIFs) were computed to detect the multicollinearity of predictors, with VIF = 1 suggesting no multicollinearity, VIF > 1 but <3 suggesting weak multicollinearity, VIF > 3 but <5 suggesting moderate multicollinearity, and VIF > 5 suggesting high multicollinearity. Generally, predictors with VIF < 5 are considered appropriate to include in regression analyses.

## 3. Results

### 3.1. Participant Demographics

Table 1 shows the demographic information for the 68 participants included in the analyses. The numbers of participants included for a specific characteristic are included under “N,” accounting for instances a participant did not provide a response. Of the 37 SSD/AHL participants, 12 had SSD, and 25 were categorized as AHL.

The traditional bilateral and SSD/AHL participants were compared on age, duration of hearing loss, SES, and sex. SES was significantly different, with higher SES in the SSD/AHL group (*p* = 0.005). Duration of hearing loss demonstrated a trend toward being longer in the traditional bilateral group than the SSD/AHL group, but there were no significant differences.

### 3.2. Preoperative Speech Recognition Performance

**Figure 1:**
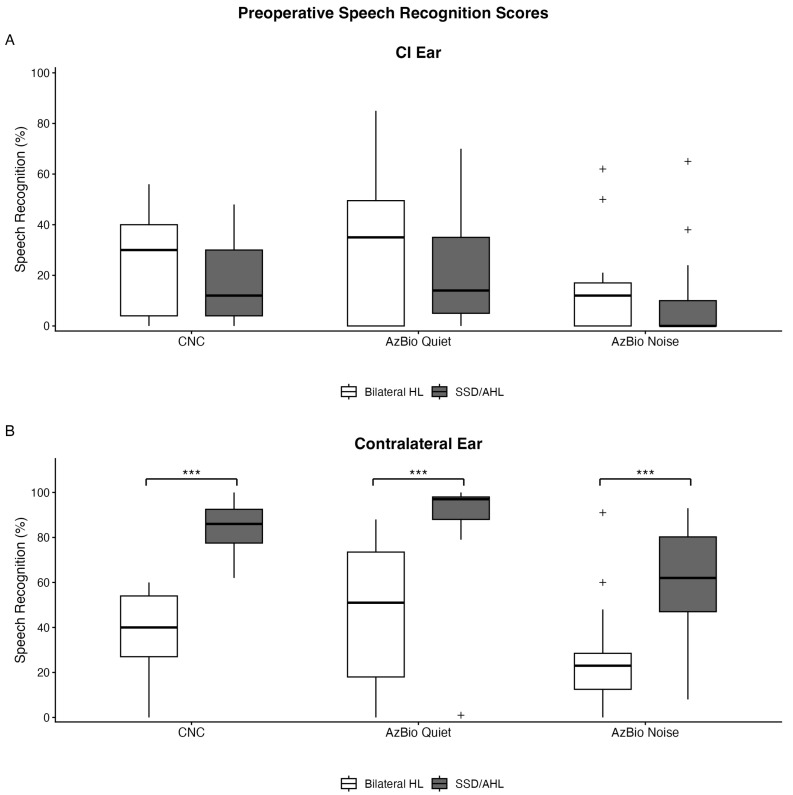
Pre-cochlear implant distribution of speech recognition scores for the (**A**) ear-to-be-implanted (CI ear) and (**B**) the non-implant ear (contralateral ear). Boxplots display CNC scores, AzBio scores in quiet, and AzBio scores in noise for each ear. The horizontal line within each box represents the median; the box spans the interquartile range (Q1–Q3). Whiskers extend to the most extreme values within 1.5 × IQR of the quartiles, and points plotted as “+” represent values outside this range (outliers). Test statistics, effect sizes, and *p*-values are reported in Table 2. Significant differences are indicated as follows: *** = *p* < 0.001.

Speech recognition performance in the CI ear did not significantly differ between the traditional bilateral hearing loss group and the SSD/AHL group. CNC word recognition scores for the CI ear were not significantly different between groups (Mann–Whitney, W = 659.5, r = 0.13, *p* = 0.289). Similarly, AzBio sentence recognition scores did not significantly differ between groups in quiet (Mann–Whitney, W = 646, r = 0.11, *p* = 0.371) or in noise (Mann–Whitney, W = 206.5, r = 0.25, *p* = 0.140) for the CI ears. See Figure 1A and Table 2 for the CNC and AzBio score distributions for the CI ear.

However, baseline speech recognition performance in the contralateral ear significantly differed between traditional bilateral hearing loss candidates and those with SSD/AHL. CNC scores for the contralateral ear were significantly higher for the SSD/AHL candidates compared to the bilateral hearing loss candidates (Mann–Whitney, W = 0, r = 0.86, *p* < 0.001). Similarly, AzBio sentence recognition scores were significantly higher for the SSD/AHL group compared to the bilateral hearing loss group in quiet (Mann–Whitney, W = 50.5, r = 0.77, *p* < 0.001) and in noise (Mann–Whitney, W = 94.5, r = 0.58, *p* < 0.001) for the contralateral ears. Each of these speech recognition differences in the contralateral ear remained significant after Holm–Bonferroni correction. See Figure 1B and Table 2 for the CNC and AzBio score distributions for the contralateral ear.

### 3.3. Preoperative PROMs

#### 3.3.1. SSQ-12

**Figure 2:**
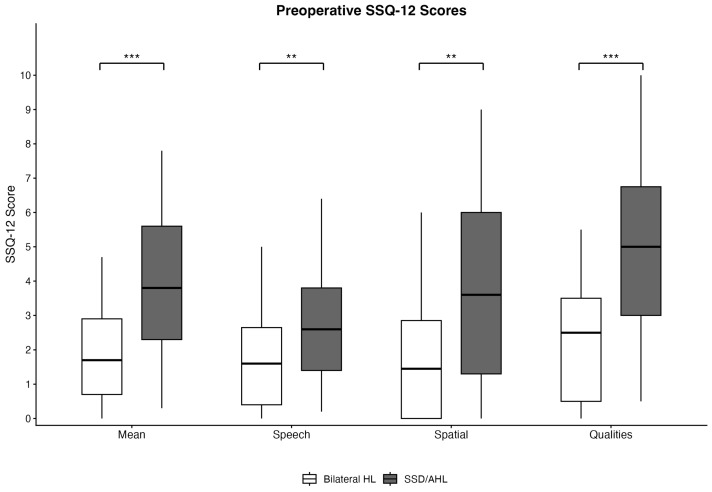
Pre-cochlear implant distribution of SSQ-12 scores. Boxplots display the distribution of mean scores within the Speech, Spatial, and Qualities domains. The distribution of the mean total score is also included. The horizontal line within each box represents the median; the box spans the interquartile range (Q1–Q3). Whiskers extend to the most extreme values within 1.5 × IQR of the quartiles. Test statistics, effect sizes, and *p*-values are reported in Table 3. Significant differences are indicated as follows: ** = *p* < 0.01, *** = *p* < 0.001.

Significant differences were observed between groups on scores across all domains of the Speech, Spatial and Qualities of Hearing Scale (SSQ-12). SSD/AHL CI candidates reported significantly higher overall preoperative SSQ-Mean scores compared to bilateral HL CI candidates (*t*-test, t = −4.23, df = 63.4, d = 1.03, *p* < 0.001). Regarding subscales of the SSQ-12, SSD/AHL participants had significantly higher preoperative SSQ-12 Speech subscale scores (Mann–Whitney, W = 320.5, r = 0.33, *p* = 0.009), SSQ-12 Spatial hearing subscale scores (Mann–Whitney, W = 300.5, r = 0.36, *p* = 0.004), and SSQ-12 Qualities subscale scores (*t*-test, t = −4.93, df = 63.7, d = 1.20, *p* < 0.001) compared to bilateral HL participants. Each of these comparisons remained significant after Holm–Bonferroni correction. See Figure 2 and Table 3 for the distribution of scores for all SSQ-12 findings.

#### 3.3.2. CIQOL-35

**Figure 3:**
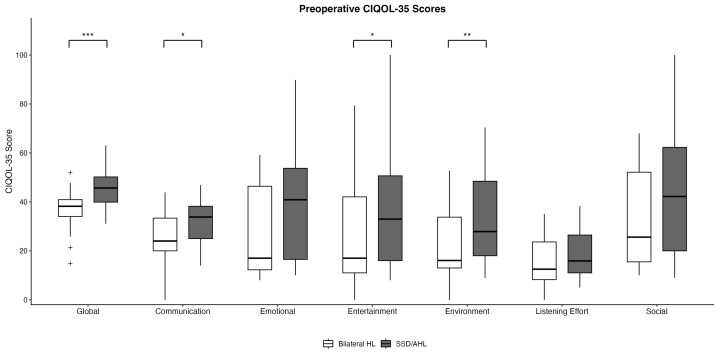
Pre-cochlear implant distribution of CIQOL-35 scores. Boxplots display the distribution of scores within the Communication, Emotional, Entertainment, Environment, Listening Effort, and Social domains. The distribution of the CIQOL-10 Global scores is also included. The horizontal line within each box represents the median; the box spans the interquartile range (Q1–Q3). Whiskers extend to the most extreme values within 1.5 × IQR of the quartiles, and points plotted as “+” represent values outside this range (outliers). Test statistics, effect sizes, and *p*-values are reported in Table 3. Significant differences are indicated as follows only when surviving Holm–Bonferroni correction: * = *p* < 0.05, ** = *p* < 0.01, *** = *p* < 0.001.

Scores on several, but not all, domains of the CIQOL-35 instrument differed significantly between groups. SSD/AHL candidates had a significantly higher CIQOL-Global score compared to bilateral HL candidates (Mann–Whitney, W = 204.5, r = 0.52, *p* < 0.001). Additionally, SSD/AHL candidates scored significantly higher than bilateral HL candidates in the following domains of the CIQOL-35: Communication (Mann–Whitney, W = 338.5, r = 0.31, *p* = 0.014), Emotional (Mann–Whitney, W = 362.5, r = 0.27, *p* = 0.033), Entertainment (Mann–Whitney, W = 328.5, r = 0.30, *p* = 0.016), and Environment (Mann–Whitney, W = 298.5, r = 0.37, *p* = 0.003). After Holm–Bonferroni correction, the CIQOL-Global, Communication, Entertainment, and Environment domains remained significantly different, while the Emotional domain did not survive Holm–Bonferroni correction. In contrast, differences were not statistically significant in the Listening Effort (Mann–Whitney, W = 411.5, r = 0.19, *p* = 0.136) and Social (Mann–Whitney, W = 398, r = 0.21, *p* = 0.096) domains. See Figure 3 and Table 3 for the distribution of scores for all CIQOL-35 findings.

### 3.4. PHQ-2, THI, and IADL

**Figure 4:**
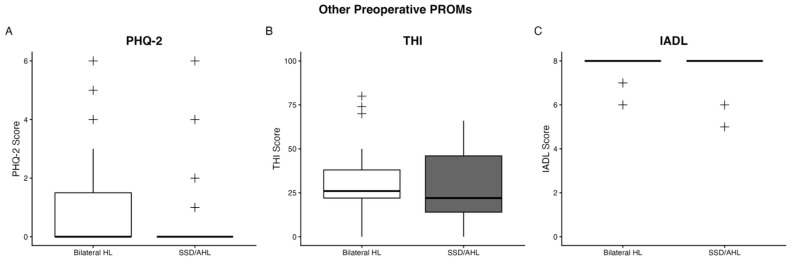
Pre-cochlear implant distribution of (**A**) PHQ-2, (**B**) THI, and (**C**) IADL scores. Boxplots display the distribution of scores for these three separate questionnaires. The horizontal line within each box represents the median; the box spans the interquartile range (Q1–Q3). Whiskers extend to the most extreme values within 1.5 × IQR of the quartiles, and points plotted as “+” represent values outside this range (outliers). Test statistics, effect sizes, and *p*-values are reported in Table 3.

PHQ-2 scores showed no significant difference between SSD/AHL and bilateral HL candidates (Mann–Whitney, W = 625, r = 0.10, *p* = 0.409). THI scores also did not significantly differ between groups (*t*-test, t = 0.61, df = 30.2, d = 0.19, *p* = 0.545), nor did IADL scores (Mann–Whitney, W = 514, r = 0.13, *p* = 0.303). See Figure 4 and Table 3 for the distribution of scores for all PHQ-2, THI, and IADL findings.

### 3.5. Relationships of CI-Ear and Contralateral-Ear CNC Scores with SSQ-Mean and CIQOL-Global

#### 3.5.1. CNC Scores and SSQ-Mean

A multivariable linear regression analysis was performed to examine the SSQ-Mean as the outcome, with aided CNC scores in the CI ear and the contralateral ear as predictors, while accounting for demographic variables of age, duration of hearing loss, and SES as additional predictors. This model was significant (R^2^ = 0.36, adjusted R^2^ = 0.29, *p* = 0.001). Only the contralateral-ear CNC score was a significant independent predictor (standardized β = 0.48, *p* = 0.002). See Table 4 for results of the analysis.

#### 3.5.2. CNC Scores and CIQOL-Global

A similar multivariable linear regression analysis was performed to examine CIQOL-Global as the outcome, with the same set of predictors as above. This model was significant (R^2^ = 0.40, adjusted R^2^ = 0.33, *p* < 0.001). Only the contralateral-ear CNC score was a significant independent predictor (standardized β = 0.62, *p* < 0.001). See Table 4 for results of the analysis.

## 4. Discussion

This study compared baseline speech recognition performance and patient-reported measures between traditional bilateral HL CI candidates and those CI candidates with SSD or AHL. Consistent with our hypothesis, traditional bilateral CI candidates demonstrated significantly worse baseline speech recognition in the contralateral ear, worse HRQOL on several domains of the CIQOL-35, and worse perceived hearing abilities on the SSQ-12. In contrast, the two groups demonstrated no significant difference in levels of depressed mood/anhedonia, tinnitus burden, and functional ability in daily activities. Together, these findings underscore the value of both audiometric measures and patient-reported measures in CI candidates for understanding baseline function and for pre-CI counseling.

### 4.1. Interpretation of Speech Recognition Measures

Baseline CNC and AzBio (in noise and quiet) speech recognition scores did not significantly differ in the CI ear between bilateral hearing loss and SSD/AHL candidates. By design, CI candidacy criteria require poor performance in the ear to be implanted (aided CNC ≤ 60%), which should equalize scores between groups when evaluating that ear in isolation. In the contralateral ear, as expected, baseline CNC and AzBio (in quiet and in noise) scores were significantly greater in SSD/AHL candidates compared to bilateral hearing loss candidates across all three measures, with large effect sizes (CNC: r = 0.86; AzBio in quiet: r = 0.77; AzBio in noise: r = 0.58). What is clinically meaningful is not the CI-ear equivalence, but rather the significant difference in contralateral ear function between the two groups. A traditional bilateral candidate is living in a fundamentally different acoustic world from an SSD/AHL candidate—even if both have comparable scores in the ear-to-be-implanted. Taken together, these findings underscore a critical limitation of ear-specific monaural testing as the sole basis for CI candidacy determination: while such testing may be sufficient for identifying an ear that may warrant implantation, it does not capture the broader auditory experience of patients, including the functional advantages of having one ear that still hears relatively well. This distinction has direct implications for counseling: traditional bilateral candidates are navigating daily life with two poorly hearing ears while SSD/AHL candidates are compensating from a position of relative asymmetric advantage. These findings therefore provide supporting evidence that traditional speech recognition measures, when limited to the CI ear alone, do not fully capture the functional differences in everyday listening experienced by the two populations.

### 4.2. Interpretation of Patient-Reported Outcome Measures

Patient-reported perceptions of hearing abilities measured by the SSQ-12 differed significantly between groups across all three subscales and overall SSQ-Mean scores. Individuals in the bilateral HL group reported substantially lower functioning related to speech understanding, spatial hearing, and sound quality in everyday environments. Starting with the Speech perception domain, bilateral HL candidates reported substantially worse scores compared to SSD/AHL candidates. This is consistent with the known impact of bilateral HL on speech intelligibility, particularly in challenging listening environments such as background noise, competing speakers, or reverberant settings [54,55,56]. SSD/AHL candidates, by contrast, retain a relatively intact ear and therefore continue to access meaningful speech information in many everyday contexts—a functional advantage that bilateral candidates lack [26]. Even larger between-group differences were observed in the Spatial hearing domain and the Qualities domain. The substantial spatial hearing deficit reported by traditional bilateral candidates is consistent with the known degradation of binaural processing that accompanies bilateral HL, including impaired access to interaural level and timing difference cues necessary for sound localization and speech-in-noise performance [57,58,59,60]. In contrast, SSD/AHL candidates retain at least one relatively functional ear and thus preserve some degree of binaural input [61,62]. The Qualities domain, which encompasses listening effort, naturalness of sound, and ease of auditory attention, was also markedly worse in the bilateral HL group, suggesting that the pervasive cognitive and attentional demands of living with bilateral HL may extend beyond simple speech understanding. Additionally, having relatively greater functional acoustic hearing in the contralateral ear supports greater naturalness of sound, as demonstrated in studies of bimodal listeners (i.e., those who use a CI on one ear and a hearing aid on the other ear) and SSD CI users [63,64,65]. These differences between groups are clinically important because they reflect distinct burdens that may not only motivate CI consideration differently in each population but may also shape post-implant benefit expectations and satisfaction trajectories. Additionally, these differences in our study also approximate established thresholds of clinical significance. A 1-point difference on the SSQ has been suggested as clinically meaningful [47,66], and the between-group differences observed in the present study were on the order of approximately 1–2.5 points across SSQ domains—with SSQ-Mean differences of almost 2 points. These findings reflect pervasive impairment in speech understanding, spatial hearing, and sound quality that affects the daily communication of bilateral HL candidates to a substantially greater degree than SSD/AHL candidates.

Several domains of the CIQOL-35 also differed significantly between groups, with SSD/AHL candidates scoring higher—reflecting better HRQOL—compared to bilateral HL candidates. Significant differences were found in the Global score, as well as in the Communication, Entertainment, and Environment domains. The Communication domain difference is consistent with the overall pattern found with the SSQ-12: bilateral candidates, lacking reliable access from either ear, may face pervasive conversational breakdowns that SSD/AHL candidates do not experience to the same degree. The Entertainment domain may reflect a similar pattern as the Communication domain and SSQ-12 findings—bilateral HL could compromise the ability to enjoy entertainment-related media without proper hearing in both ears, whereas SSD/AHL allows for preservation of enough hearing function to interact with entertaining media. The Environment domain, which captures awareness of environmental and warning sounds, similarly favors SSD/AHL candidates, consistent with their retention of one functional ear for passive monitoring of the acoustic environment. Additionally, traditional bilateral HL candidates self-report significantly worse overall HRQOL than SSD/AHL candidates on the CIQOL Global scale. These findings suggest that the two groups should be considered differently when it comes time for preoperative counseling so that patients can have better expectations for how the CI might benefit them or not. Furthermore, the CIQOL manual provides values for the conditional minimum detectable change (cMDC) as a benchmark of clinical significance, ranging from approximately 10 to 18 points depending on the CIQOL domain for our patient’s data [49]. The statistically significant between-group differences (bilateral HL vs. SSD/AHL) on the CIQOL observed in this study approximate these thresholds, suggesting that the group-level differences are not only statistically meaningful but also clinically relevant.

In contrast, non-significant group differences were found for the CIQOL-35 Emotional, Social, and Listening Effort domains. These findings could relate to fact that the psychological and social impacts of hearing loss are influenced by a more complex interplay of personality, social support, communication strategy use, and environmental accommodation that may attenuate the direct effects of hearing loss configuration [67,68,69]. Additionally, the finding that Listening Effort did not differ significantly between groups is intriguing, as one might expect traditional bilateral candidates to report greater effort given their more severe hearing-loss profile. Given that their Listening Effort domain scores were overall low (i.e., participants reported relatively high listening effort), this convergence may reflect a “floor effect” in which both groups experience substantial listening effort in everyday environments due to having at least one poorly functioning ear, albeit potentially from different mechanisms—binaurally poor input in the bilateral group versus the cognitive effort of compensating for absent input from one ear in SSD/AHL. This idea that any form of hearing impairment might lead to high levels of listening effort has been explored in the past with various levels of hearing loss, but there need to be additional studies comparing these populations specifically as they become more distinctly recognized [70,71]. Alternatively, the sample size of this study may have limited statistical power to detect true differences in these domains.

Interestingly, broader psychosocial measures—including depressive symptom screening (PHQ-2), tinnitus burden (THI), and functional independence (IADL)—did not differ significantly between groups. These findings indicate that although hearing-specific functional limitations differ between bilateral hearing loss and SSD/AHL populations, broader measures of psychological well-being and daily functioning may be similar. The psychological sequelae of hearing loss—at least as captured by a brief depression screen—did not differ meaningfully based on hearing loss configuration in this older adult sample. While rates of depression are elevated in adults with hearing loss relative to the general population, the severity and laterality of hearing impairment may not be the primary driver of depressive symptoms at the point of CI candidacy evaluation [38,72]. Factors such as social isolation, communication difficulty, and loss of valued activities, which can occur across the spectrum of hearing loss, may contribute more uniformly to the affective burden regardless of whether the hearing loss is bilateral or asymmetric [73,74]. The similar tinnitus burden between groups also is consistent with the literature, suggesting that tinnitus is common across hearing loss configurations and not reliably predicted by audiometric severity or symmetry [75,76]. Of note, both groups demonstrated mean THI scores in the “mild” severity range. However, the range of THI scores was quite broad for both groups, with a subset of patients having tinnitus in the moderate or severe range. The high IADL scores in both groups indicate that these CI candidates are largely independent in their daily living activities.

Taken together, these results suggest that objective audiologic testing alone may underestimate meaningful differences in the everyday listening experiences of CI candidates. PROMs provide valuable complementary information and may be particularly useful when evaluating individuals with asymmetric auditory configurations.

### 4.3. Relations of CNC Scores with SSQ-Mean and CIQOL-Global

The regression analyses revealed a consistent and clinically informative dissociation between CI-ear and contralateral-ear CNC performance in their relationships with patient-reported hearing ability (SSQ-Mean) and HRQOL (CIQOL-Global) in our cohort. Across both measures, CI-ear CNC scores were not significantly associated with the outcomes, while contralateral-ear CNC scores demonstrated moderate and statistically significant associations. These findings support our hypothesis that pre-implant, contralateral-ear CNC scores would show a stronger relationship with PROMs than CNC scores for the CI ear.

The absence of meaningful associations between CI-ear CNC performance and SSQ-Mean or CIQOL-Global scores in pre-CI participants aligns with prior studies examining post-CI outcomes showing similar weak associations [17,32,33,34]. It is plausible that when one ear performs poorly enough to warrant implantation, its objective performance may have a weaker relationship with how patients perceive their overall hearing abilities and HRQOL—with the subjective experience potentially shaped more by what the better-functioning ear can or cannot provide. This interpretation appears to be consistent with the broader literature on binaural hearing, which indicates that the daily listening experience in individuals with asymmetric hearing configurations is strongly influenced by the input from the better ear [43,77]. It should be noted, however, that because the data from our study are based on monaural (not binaural), ear-specific CNC testing, the current study does not directly assess bilateral listening or real-world ear dominance. Future studies incorporating bilateral test conditions would be needed to more fully evaluate this explanation. Additionally, it should be acknowledged that the larger (i.e., moderate-sized) contralateral-ear CNC associations with the SSQ-Mean and CIQOL-Global observed may also be partially attributable to the broader range of contralateral-ear CNC performance introduced by including both bilateral hearing loss and SSD/AHL participants together in analyses.

Taken together, findings of this study have implications for how pre-implant audiologic assessment might be interpreted in the context of patient counseling. The contralateral-ear CNC score—which is routinely collected during CI candidacy evaluation but has received limited attention as a predictor of patient-reported outcomes—may carry useful information about a candidate’s functional hearing abilities or limitations and HRQOL beyond what the CI-ear score alone conveys. Clinicians may therefore find value in considering the contralateral-ear score not only as a criterion for categorizing the candidacy type (bilateral loss vs. SSD/AHL), but as an independent source of information about a patient’s subjective hearing experience that can inform expectation-setting conversations prior to implantation.

### 4.4. Clinical Implications

The findings of this study suggest several practical implications for clinicians involved in the evaluation and counseling of adult CI candidates. First, the divergence between findings for CI-ear speech recognition scores and patient-reported hearing abilities underscores a limitation of relying solely on audiometric testing. Specifically, CI-ear CNC scores, which serve as the primary criterion for determining candidacy and categorizing hearing loss type, showed no significant association with the SSQ-Mean or CIQOL-Global in this sample. This suggests that the measure used to qualify a patient for implantation carries little information about how that patient perceives their hearing abilities and HRQOL. Administering PROMs such as the SSQ-12 and CIQOL-35 as part of routine preoperative evaluation can therefore help clinicians understand the dimensions of a patient’s hearing experience that the candidacy-defining audiometric measure does not capture, and this information could be leveraged to set more realistic and individualized post-implant expectations.

Second, the substantially different self-reported hearing abilities and HRQOL between SSD/AHL and traditional bilateral candidates at baseline suggests that their starting point for CI benefit may be meaningfully different. Prior work has demonstrated that higher pre-CI baseline scores on HRQOL measures are associated with smaller post-CI gains, as there is less room for improvement relative to those starting from a lower baseline [78,79]. Clinicians could therefore counsel SSD/AHL patients that while CI may improve specific aspects of their hearing (e.g., sound localization, hearing in noise from the implanted side, and tinnitus suppression), the magnitude of change on broad HRQOL measures may be more modest than for traditional bilateral patients, though future research studies are needed to confirm this. Conversely, traditional bilateral candidates—who report significantly worse baseline HRQOL and hearing-specific functional difficulties—may stand to achieve larger absolute gains across multiple HRQOL domains following implantation, an encouraging message to share during counseling.

Third, the finding that psychosocial and global daily independence measures (the PHQ-2, THI, and IADL) did not differ between groups implies that baseline emotional and functional status may not need to be counseled differently based on hearing loss configuration—at least for older adults in the age range of this study. However, clinicians should remain attentive to individual patients who fall outside the group averages, particularly those with elevated PHQ-2 or THI scores, as these factors have been independently associated with post-CI satisfaction and decision regret in prior studies [11,13]. The uniformly high IADL scores across both groups also suggest that the CI candidate population evaluated here represents a relatively high-functioning older adult cohort, and caution should be exercised in generalizing these findings to patients with a greater comorbidity burden or cognitive impairment.

Based on these findings, we suggest the following actionable clinical recommendations: (1) Administer the SSQ-12 and CIQOL-35 as part of routine pre-CI evaluation for all candidates, regardless of hearing loss configuration, to capture HRQOL that audiometric testing alone does not reflect. (2) Counsel SSD/AHL patients that while CI may improve specific listening challenges (e.g., sound localization, hearing from the poorer side, and tinnitus suppression), large gains on broad HRQOL measures may be less likely given their higher baseline. (3) Counsel traditional bilateral candidates that their substantially lower HRQOL baseline affords greater room for improvement across multiple HRQOL domains post-implantation.(4) Integrate the contralateral-ear CNC score—routinely collected but underutilized—into preoperative counseling conversations as a proxy for a patient’s subjective hearing experience and daily listening function.

### 4.5. Limitations and Future Directions

One limitation of the present study is the grouping of SSD and AHL into a single SSD/AHL category because the number of participants with SSD was relatively small (*n* = 12), which would have limited the ability to draw robust conclusions for that subgroup alone. Although these populations share a similar reliance on a better-hearing ear, they represent distinct clinical conditions and may differ in their functional and HRQOL impacts. Readers should interpret the results with awareness that heterogeneity within the SSD/AHL group may attenuate or obscure subgroup differences. Additionally, some patient groups were explicitly excluded. For example, patients with retrocochlear pathologies (e.g., vestibular schwannoma) were excluded, for whom pre- or intra-operative electrophysiological testing of auditory nerve integrity might be a valuable factor to consider [80,81]. Future studies with more diverse etiologies and larger sample sizes could compare similar speech recognition measures and PROMs among AHL, SSD, and traditional bilateral hearing loss as three separate groups. This would allow for even greater understanding of the differences in pre-CI baseline HRQOL and PROMs for different CI populations.

Second, the present study focuses solely on preoperative measures. Longitudinal studies examining similar speech recognition measures and HRQOL for pre-CI candidates compared with their post-CI measures for AHL, SSD, and traditional bilateral hearing loss are necessary. This approach would provide important information about how these abilities and perceptions change over time in these three CI candidate groups, so that clinicians can set more accurate and realistic expectations for individual CI recipients. Additionally, this study reports cross-sectional, preoperative data only. Without postoperative follow-up, we cannot determine whether the group differences observed at baseline persist, diminish, or reverse after cochlear implantation. Our hope is that a future study will expand on these findings by evaluating preoperative and postoperative speech recognition measures and PROMs for these three groups (traditional bilateral hearing loss, AHL, and SSD).

Third, the study sample was recruited from a single tertiary referral CI center and consists predominantly of older adults with relatively high educational attainment, high SES, and near-maximal IADL scores. This profile limits the generalizability to broader CI candidate populations, including younger adults, those with lower SES, or those with a greater comorbidity burden. Readers and clinicians should exercise caution when extrapolating these findings to populations that differ demographically from the cohort studied here.

Fourth, several potential confounding variables were not systematically controlled, including the etiology of hearing loss, duration and type of hearing aid use, and cognitive status beyond exclusion of diagnosed impairment. These uncontrolled variables could influence the HRQOL, PROMs and the relationships observed between CNC scores.

Fifth, the inherent subjectivity of self-report measures represents a limitation. PROMs are susceptible to response bias, social desirability effects, and variability in how individuals interpret questionnaire items—none of which can be fully mitigated by standardized administration. Nevertheless, these PROMs are the best tools we currently have for assessing and comparing the subjective experiences of CI candidates and users.

Sixth, speech recognition was assessed only in quiet and noise conditions in the clinic; real-world listening environments are considerably more varied and complex, and performance in these controlled conditions likely does not fully reflect patients’ functional hearing in daily life.

Seventh, given that statistical significance at moderate effect sizes with the current sample sizes (*n* = 31 bilateral HL; *n* = 37 SSD/AHL) does not guarantee clinical meaningfulness, and given that non-significant findings do not imply equivalence—particularly for comparisons where power was limited—all group comparisons should be interpreted with appropriate caution and contextualized by effect sizes.

Finally, the currently available literature on cochlear implantation outcomes in bilateral hearing loss, SSD, and AHL remains limited, with few studies directly comparing these three candidate groups. Consequently, the extent to which the present findings can be contextualized within or corroborated by existing research is constrained. These results should therefore be interpreted with appropriate caution and considered alongside future studies to confirm the generalizability of our findings and expand the evidence base for these CI candidacy populations.

## 5. Conclusions

This study characterized and compared preoperative speech recognition and PROMs between traditional bilateral hearing loss and SSD/AHL CI candidates. Although CI-ear speech recognition was not significantly different between groups, contralateral-ear performance was significantly better in SSD/AHL candidates, while bilateral candidates reported worse hearing-specific abilities and HRQOL across most domains. In contrast, depressive symptoms, tinnitus burden, and functional independence did not significantly differ, suggesting that group disparities are hearing-specific rather than reflective of overall health. Additionally, this study found that CI-ear CNC performance was not significantly associated with the SSQ-Mean or CIQOL-Global, whereas contralateral-ear CNC performance showed moderate relationships. These findings suggest that the ear used to determine candidacy does not fully reflect patients’ lived auditory experiences, and that contralateral-ear function likely provides clinically relevant information that may further enhance counseling. Together, these results support the relevance and importance of baseline PROMs, HRQOL, and contralateral-ear measures in preoperative evaluation of CI candidates to further determine how to improve individualized counseling and expectation setting during the pre-implant period.

## Figures and Tables

**Table 1:** Demographic information for included participants.

			N	Value	
**Age (years)**			**68**		
Range				55–86	
Mean ± SD				71.6 ± 7.4	
**Sex**			**68**		
Male				42	
Female				26	
**Type of Hearing Loss**			**68**		
SSD/AHL				37	
Traditional bilateral				31	
**Duration of Hearing Loss (years)**			**67**		
Range				0–76	
Mean ± SD				22.8 ± 18.6	
**Highest Level of Education**			**64**		
Did not complete high school				1	
High school graduate or equivalent				17	
Some college/trade/technical/vocational training				5	
Associate degree				3	
Bachelor’s degree				16	
Master’s degree or higher				22	
**Employment Status**			**67**		
Employed				16	
Not employed				2	
Retired				49	
**Socioeconomic Status**			**56**		
Range				3.5–64	
Mean ± SD				29.4 ± 17.5	
**Demographic Comparisons between Traditional Bilateral HL vs. SSD/AHL**					
	**Bilateral HL** **(Mean ± SD)**	**SSD/AHL (Mean ± SD)**	**Test Statistic**	**Effect Size**	***p*** **-value**
**Age**	72.45 ± 7.44	70.92 ± 7.39	t = 0.85, df = 63.8	d = 0.21	0.399
**Duration of HL**	27.8 ± 20.36	18.81 ± 16.28	W = 708.5	r = 0.24	0.054
**SES**	23.52 ± 18.23	35.25 ± 14.93	W = 223	r = 0.37	**0.005**
**Sex**	12 F, 19 M	14 F, 23 M	χ^2^ < 0.001	Cramer’s V = 0.01	1

SD indicates standard deviation; SSD, single-sided deafness; AHL, asymmetric hearing loss; HL, hearing loss; SES, socioeconomic status; t, test statistic for *t*-test; df, degrees of freedom; W, Wilcoxon rank-sum statistic (from Mann–Whitney U test); d, Cohen’s d; r, Pearson correlation coefficient; F, female; M, male.

**Table 2:** Comparison of baseline speech recognition scores.

	Bilateral HL (Mean ± SD)	SSD/AHL (Mean ± SD)	Test Statistic	Effect Size	*p*-Value
**Ear-to-be-implanted (CI ear)**					
CNC score (%)	23.48 ± 18.79	17.78 ± 17.28	W = 659.5	r = 0.13	0.289
AzBio score in quiet (%)	29.74 ± 26.30	22.49 ± 21.57	W = 646	r = 0.11	0.371
AzBio score in noise (%)	14 ± 17.67	9.63 ± 16.95	W = 206.5	r = 0.25	0.140
**Contralateral ear (non-CI ear)**					
CNC score (%)	38.13 ± 18.83	83.69 ± 11.25	W = 0	r = 0.86	**<0.001**
AzBio score in quiet (%)	46.94 ± 28.89	90.55 ± 17.88	W = 50.5	r = 0.77	**<0.001**
AzBio score in noise (%)	26.35 ± 20.93	59.6 ± 23.47	W = 94.5	r = 0.58	**<0.001**

HL indicates hearing loss; SSD/AHL, single-sided deafness/asymmetric hearing loss; CNC, Consonant–Nucleus–Consonant; SD, standard deviation; W, Wilcoxon rank-sum statistic (from Mann–Whitney U test); r, Pearson correlation coefficient.

**Table 3:** Statistical analysis of preoperative patient-reported outcome measures.

	Bilateral HL (Mean ± SD)	SSD/AHL (Mean ± SD)	Test Statistic	Effect Size	*p*-Value
**SSQ-12**					
Mean	1.96 ± 1.40	3.71 ± 1.97	t = −4.23, df = 63.4	d = 1.03	**<0.001**
Speech Perception	1.72 ± 1.44	2.84 ± 1.72	W = 320.5	r = 0.33	**0.009**
Spatial Hearing	1.84 ± 1.91	3.74 ± 2.66	W = 300.5	r = 0.36	**0.004**
Qualities Domain	2.25 ± 1.70	4.70 ± 2.33	t = −4.93, df = 63.7	d = 1.20	**<0.001**
**CIQOL-35**					
Global	36.65 ± 7.45	45.36 ± 7.51	W = 204.5	r = 0.52	**<0.001**
Communication	24.71 ± 11.25	31.71 ± 9.17	W = 338.5	r = 0.31	**0.014**
Emotional	28.18 ± 18.17	36.67 ± 21.38	W = 362.5	r = 0.27	0.033 *
Entertainment	24.98 ± 19.76	37.89 ± 24.35	W = 328.5	r = 0.30	**0.016**
Environment	21.73 ± 13.46	33.50 ± 18.08	W = 298.5	r = 0.37	**0.003**
Listening Effort	14.86 ± 9.54	18.45 ± 9.61	W = 411.5	r = 0.19	0.136
Social	32.12 ± 18.66	42.87 ± 27.23	W = 398	r = 0.21	0.096
**PHQ-2**	0.94 ± 1.71	0.57 ± 1.37	W = 625	r = 0.10	0.409
**THI**	32.82 ± 23.2	28.67 ± 19.82	t = 0.61, df = 30.2	d = 0.19	0.545
**IADL**	7.80 ± 0.55	7.86 ± 0.59	W = 514	r = 0.13	0.303

HL indicates hearing loss; SSD/AHL, single-sided deafness/asymmetric hearing loss; SD, standard deviation; SSQ-12, Speech, Spatial and Qualities of Hearing Scale; CIQOL-35, Cochlear Implant Quality of Life–35; PHQ-2, Patient Health Questionnaire-2; THI, Tinnitus Handicap Inventory; IADL, Instrumental Activities of Daily Living; t, test statistic for *t*-test; df, degrees of freedom; W, Wilcoxon rank-sum statistic (from Mann–Whitney U test); d, Cohen’s d; r, Pearson correlation coefficient. * Did not survive the Holm–Bonferroni correction, so this *p*-value is not significant even though it is <0.05.

**Table 4:** Linear regression analysis involving CI-ear and contralateral-ear CNC scores with the SSQ-Mean and CIQOL-Global.

Predictors	Unstandardized B	Coefficient SE	Standardized β Coefficient	t	*p*-Value	VIF
**Dependent measure: SSQ-Mean**						
Age	−0.047	0.034	−0.18	−1.40	0.167	1.140
Duration of HL	0.021	0.014	0.21	1.52	0.135	1.263
SES	0.022	0.015	0.21	1.49	0.144	1.327
CNC Score—CI ear	0.011	0.013	0.11	0.82	0.419	1.132
CNC Score—contralateral ear	0.033	0.010	0.48	3.28	**0.002**	1.465
**Dependent measure: CIQOL-Global**						
Age	0.048	0.155	0.04	0.31	0.758	1.136
Duration of HL	0.052	0.064	0.11	0.82	0.420	1.274
SES	0.045	0.069	0.09	0.66	0.516	1.321
CNC Score—CI ear	0.036	0.062	0.07	0.58	0.568	1.138
CNC Score—contralateral ear	0.201	0.047	0.62	4.31	**<0.001**	1.441

SE indicates standard error; t, t-value; VIF, Variance Inflation Factor; HL, hearing loss; SES, socioeconomic status; CNC, Consonant–Nucleus–Consonant; CI, cochlear implant; SSQ, Speech, Spatial and Qualities of Hearing Scale; CIQOL, Cochlear Implant Quality of Life.

## Data Availability

The raw data supporting the conclusions of this article may be made available by the authors on request.

## Abbreviations

The following abbreviations are used in this manuscript:

CI	Cochlear implant
SSD	Single-sided deafness
AHL	Asymmetric hearing loss
PROM	Patient-reported outcome measure
CNC	Consonant–Nucleus–Consonant
SSQ	Speech, Spatial and Qualities of Hearing Scale
CIQOL	Cochlear Implant Quality of Life
PHQ	Patient Health Questionnaire
THI	Tinnitus Handicap Inventory
IADL	Instrumental Activities of Daily Living
WHO	World Health Organization
HRQOL	Hearing-related quality of life
SD	Standard deviation
SE	Standard error
MSTB	Minimum Speech Test Battery
VIF	Variance Inflation Factor
HL	Hearing loss
